# A single origin and high genetic diversity of cultivated medicinal herb *Glehnia littoralis subsp*. *littoralis* (Apiaceae) deciphered by SSR marker and phenotypic analysis

**DOI:** 10.1371/journal.pone.0308369

**Published:** 2024-08-08

**Authors:** Weiwei Li, Shuliang Liu, Shimeng Wang, Yihui Li, Dongrui Kong, Ailan Wang

**Affiliations:** School of Life Sciences, Ludong University, Yantai, Shandong, China; KGUT: Graduate University of Advanced Technology, ISLAMIC REPUBLIC OF IRAN

## Abstract

Ten SSR markers based on transcriptome sequencing were employed to genotype 231 samples of *G*. *littoralis subsp*. *littoralis* (Apiaceae) from nine cultivated populations and seven wild populations, aiming to assess the genetic diversity and genetic structure, and elucidate the origin of the cultivated populations. Cultivated populations exhibited relatively high genetic diversity (h = 0.441, I = 0.877), slightly lower than that of their wild counterparts (h = 0.491, I = 0.930), likely due to recent domestication and ongoing gene flow between wild and cultivated germplasm. The primary cultivated population in Shandong have the crucial genetic status. A single origin of domestication was inferred through multiple analysis, and wild populations from Liaoning and Shandong are inferred to be potentially the ancestor source for the present cultivated populations. Phenotypic analysis revealed a relatively high heritability of root length across three growth periods (0.683, 0.284, 0.402), with significant correlations observed between root length and petiole length (Pearson correlation coefficient = 0.30, *P*<0.05), as well as between root diameter and leaf area (Pearson correlation coefficient = 0.36, *P<*0.01). These parameters can serve as valuable indicators for monitoring the developmental progress of medicinal plants during field management. In summary, this study can shed light on the intricate genetic landscape of *G*. *littoralis subsp*. *littoralis*, providing foundational insights crucial for conservation strategies, targeted breeding initiatives, and sustainable management practices in both agricultural and natural habitats.

## Introduction

*Glehnia littoralis* Fr. Schmidt ex Miq. *subsp*. *littoralis*, a perennial diploid (2n = 22) [1–3] herbaceous plant belonging to the Apiaceae family, holds significant botanical and medicinal importance, particularly in China, where it is known as Radix Glehniae, a traditional and highly esteemed medicinal herb [4, 5]. Radix Glehniae encompasses a plethora of bioactive compounds, including coumarins, alkaloids, and polysaccharides, which exhibit noteworthy therapeutic properties in modulating immune responses, addressing tumors, and managing cardiovascular and cerebrovascular ailments [6–14]. As a staple in clinical Chinese medicine, the annual demand for Radix Glehniae exceeds 4,000 tons in China alone, reflecting its substantial economic value. However, the native habitat of *G*. *littoralis subsp*. *littoralis*, primarily coastal mudflats, faces threats due to human activities, posing challenges to the wild population’s sustainability. Consequently, *G*. *littoralis subsp*. *littoralis* has been designated as a nationally second-class protected wild plant [15]. Presently, the primary source of medicinal materials derived from *G*. *littoralis subsp*. *littoralis* is cultivated cultivars, predominantly cultivated in provinces such as Shandong, Hebei, and Inner Mongolia, fostering a large-scale and industrialized production pattern. Notably, *G*. *littoralis subsp*. *littoralis* harvested in Laiyang City, Yantai City, Shandong Province, is known as "Laiyang shen". Due to its elevated coumarin content and superior quality, it is recognized as an authentic medicinal herb. In recent years, challenges have arisen from farmers’ inability to discern between different genotypes during cultivation, compounded by frequent gene exchange between cultivars resulting from cross-pollination and self-pollination behaviors of *G*. *littoralis subsp*. *littoralis*. Consequently, the genetic diversity and stability of *G*. *littoralis subsp*. *littoralis* have suffered, leading to a decline in the quality of the medicinal herb Radix Glehniae. The cultivated land area of *G*. *littoralis subsp*. *littoralis* in Laiyang has experienced a substantial reduction. However, Anguo City in Hebei Province and Chifeng City in Inner Mongolia have progressively emerged as the primary production areas for *G*. *littoralis subsp*. *littoralis*. Based on this, it is suspected that the authentic producing area of Radix Glehniae has been transferred from Laiyang City to other areas, resulting in a consequent decline in cultivation profits. To enhance the quality of this medicinal herb, boost farmers’ income, and advocate for the sustainable utilization of *G*. *littoralis subsp*. *littoralis*, there is an urgent need to investigate and assess the quality variations of *G*. *littoralis subsp*. *littoralis* across different regions.

The authenticity of Chinese medicinal materials has consistently been a hot issue in the quality assessment of these materials [16]. Given the extensive distribution of Chinese medicinal resources, variations in the quality of Chinese medicinal materials from different areas are often conspicuous. Distinct populations not only exhibit phenotypic differences (in chemical composition) but also diverge at the genotype level. For instance, the effective chemical composition of diverse populations of medicinal plants, such as *Curcuma longa* L. [17], *Artemisia herba-alba* Asso. [18] and *Epimedium sagittatum* (Siebold & Zucc.) Maxim. [19], is influenced by the extent of genetic variation between populations, revealing a significant correlation between genetic variation and variations in metabolic products. Unraveling the genetic differentiation among populations within the original plant species proves instrumental in elucidating the internal mechanisms contributing to the authenticity of traditional Chinese medicine. Prior genetic diversity investigations into wild *G*. *littoralis subsp*. *littoralis* have indicated that genetic distances between populations in this species do not exhibit a significant correlation with geographic distance [20, 21]. The difference of genetic distance between populations determines the difference of genetic differentiation between populations. It can be inferred that the degree of genetic differentiation between populations in different geographical regions of *G*. *littoralis subsp*. *littoralis* is more likely to be determined by genetic factors rather than environmental factors. This underscores the necessity for a comprehensive study to shed light on the genetic intricacies influencing the authenticity and quality variations of *G*. *littoralis subsp*. *littoralis* across different regions.

The water-dependent dissemination of pollen and seeds contributes to the diminished genetic differentiation among wild populations, as it facilitates a significant gene flow across extensive geographical distances [22]. Nonetheless, cultivated populations might exhibit distinct genetic structures, necessitating additional investigation. Therefore, our study delved into the genetic diversity of cultivated *G*. *littoralis subsp*. *littoralis* across various production regions, utilizing SSR molecular markers [23–25]. The primary objective was to unravel the genetic structure and differentiation within and between *G*. *littoralis subsp*. *littoralis* populations in diverse regions. Concurrently, we explored the correlation and heritability of morphological traits within cultivated *G*. *littoralis subsp*. *littoralis* populations during different growth stages. This examination aimed to assess the impact of genetic regulation on morphological traits. The research results can establish a theoretical foundation for resource conservation, development, utilization, variety selection, introduction, and cultivation of the medicinal plant *G*. *littoralis subsp*. *littoralis*.

## Materials and methods

### Samples collection

**Fig 1:**
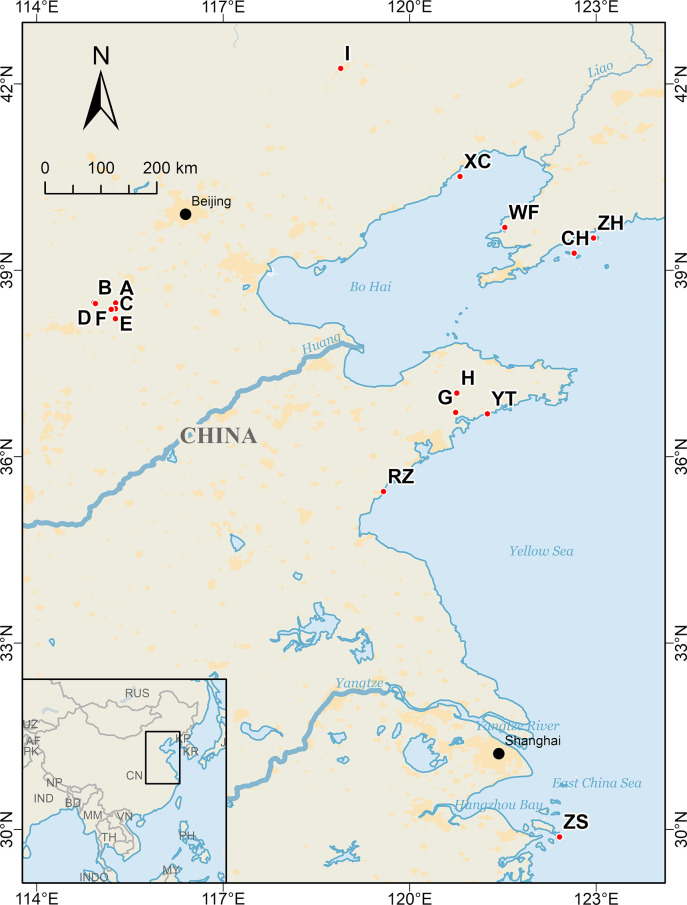
Geographic locations of 16 populations of *Glehnia littoralis subsp*. *littoralis* in this study. The red dots represent 16 population locations. A-I represent cultivated populations and YT, CH, WF, ZH, ZS, RZ, XC represent wild populations.

A total of 231 samples from 16 *G*. *littoralis subsp*. *littoralis* populations were collected, comprising nine populations in three primary cultivation regions (Shandong, Hebei, Inner Mongolia) and seven wild populations. The selection of Seven distinct wild populations was based on previous molecular marker analyses, ensuring representation of all genetic variations in wild resources [20, 21, 26]. Additionally, morphological data from cultivated *G*. *littoralis subsp*. *littoralis* in the three main production areas were gathered at three developmental stages (seedling, vigorous growth, and harvesting). Samples from the seedling stage, the vigorous stage, and the harvesting stage were collected approximately 30 days, 120 days, and 180 days after the germination of *G*. *littoralis subsp*. *littoralis* seeds, respectively. Six representative individuals were chosen for each stage to collect morphological data, encompassing root length, petiole length, root diameter, and total leaf area. All phenotypic data are detailed in S1 Table. The seeds utilized for mRNA sequencing analysis were collected from the beaches in Haiyang City, Shandong. The collection information can be found in Table 1 and Fig 1. The specimens of *G*. *littoralis subsp*. *littoralis* are stored in the School of Life Sciences, Ludong University. All materials are collected and used in accordance with relevant agencies, national guidelines and regulations, and with the permission of local environmental protection departments.

**Table 1:** All the 231 samples from 16 populations of *Glehnia littoralis subsp*. *littoralis* used in this study.

Population ID	Locations	No. of sample	Longitude	Latitude
A	Beiduancun, Hebei	26	115°16’9"	38°28’28"
B	Mingyuedian, Hebei	15	114°55’10"	38°28’50"
C	Shenze, Hebei	19	115°16’4"	38°13’16"
D	Zhoucun, Hebei	16	114°56’47"	38°27’54"
E	Dongliuchun, Hebei	17	115°16’5"	38°22’54"
F	Dawunv, Hebei	22	115°11’52"	38°22’12"
G	Gaogezhuang,Shandong	18	120°44’26"	36°42’42"
H	Muyudian,Shandong	19	120°45’34"	37°1’30"
I	Niujiaying, Inner Mongolia	12	118°53’24"	42°14’52"
YT	Haiyang, Shandong	10	121°15′	36°41′24″
CH	Changhai, Liaoning	10	122°38′47″	39°16′25″
WF	Wafangdian, Liaoning	11	121°31′48″	39°41′24″
ZH	Zhuanghe, Liaoning	10	122°57′35″	39°31′12″
ZS	Zhoushan, Zhejiang	10	122°24′36″	29°52′48″
RZ	Rizhao, Shandong	9	119°34′48″	35°26′24″
XC	Xingcheng, Liaoning	7	120°48′36″	40°30′36″

### Morphological feature measurement

After collecting plant samples, organize the leaves and roots according to specific criteria. Leaves should be arranged so that their surfaces do not overlap and lie flat. Secure them using transparent tape. To ensure optimal photo contrast, use a white background and include a ruler for scale in each image. Utilize ImageJ software for analyzing and measuring the data captured in the photographs [27]. Leaf area calculation involves fully extending each leaf and measuring its area individually. Sum these values to accurately determine the total leaf area of the plant. Root diameter is determined by measuring the width at ten evenly distributed points along the root and averaging these measurements. Root length is measured from the clear interface between roots and rhizomes to the furthest point of the main root. Petiole length assessment involves selecting the longest petioles and measuring from their connection point with the stem to the leaf base. To ensure data reliability and accuracy, each measurement except root diameter is performed three times, and the average value is used. Biological evaluation of each characteristic is conducted six times.

### Transcriptome sequencing and SSR mining

The transcriptome sequencing was conducted on the seed samples subsequent to processing. Total RNA was extracted from seed with three biological replicates using TRI Reagent (Sigma Life Science, USA), according to manufacturer’s instructions. RNA integrity was confirmed by using the Agilent 2100 Bioanalyzer. A total of 0.5–2 μg RNA per sample was sent for library preparation using the TruSeq RNA sample preparation kit (Illumina RS-122–2101, Illumina, CA, USA). The library was sequenced on an Illumina HiSeq2000 instrument. The libraries were sequenced utilizing the Illumina genome analyzer platform (Annoroad, Beijing, China), with a read length of 150 bp. The raw reads were subjected to initial processing, including removal of adapter sequences, empty reads, low-quality sequences, and all reads with base quality below Q20. Subsequently, the clean reads underwent assembly into contigs utilizing Trinity software with default parameters [28]. The raw sequencing data has been submitted to the NCBI Sequence Read Archive (SRA) database (SRR28033806- SRR28033808). MISA software (MIcroSAtellite identification tool) was employed to scour the transcriptome sequencing results and identify microsatellite loci [29]. Twenty pairs of SSR primers were designed using Primer 3 software [30, 31].

### DNA extraction and SSR amplification

Total DNA extraction was carried out using the CTAB method [32]. The concentration and purity of DNA were determined through spectrophotometry and agarose gel electrophoresis. The DNA concentration was adjusted to 30 ng/μL for subsequent SSR PCR reactions. Sixteen SSR primers were initially utilized to screen for suitable primers with relatively high polymorphism and band reproducibility. Ultimately, 10 primers were selected, and their sequences are listed in Table 2. The PCR reaction was conducted in a 15 μL reaction system, comprising 7.5 μL of 2×Taq DNA polymerase PCR master mix buffer, 1 μL of 5 mM primer, 0.5 μL of plant DNA, and 5 μL of sterile double-distilled water. The thermal cycling conditions were as follows: an initial denaturation at 95°C for 5 minutes, followed by 35 cycles of denaturation at 94°C for 30 seconds, annealing at 56°C for 30 seconds, extension at 72°C for 1 minute, and a final extension at 72°C for 5 minutes. PCR products were separated on an 8% denaturing polyacrylamide gel with electrophoresis at 150 V for a stable 120 minutes. Post-electrophoresis, silver staining was executed, and the gel was photographed, with the results being saved. The bands were counted based on clarity, length, and reproducibility, and a data matrix was formed.

**Table 2:** Characterization of 10 microsatellite loci.

Locus	Primer sequnence (5’-3’)	Repeat	T_m_ (°C)	Size (bp)
SHCSSR1	F:TGGTTTCTTTGACAAGGAAG	(CCT)5	56	127–228
	R:CATCAGAGGCAGCACATC			
SHCSSR2	F:GTGTGGCAGTGATTGTGT	(TG)11	56	120–140
	R:CAGTTAAATATCGAGCACAGC			
SHCSSR3	F:ATATACGGCATCTGAGTGAT	(TG)20	56	125–190
	R:GACCGAACTTAAGGCAATC			
SHCSSR5	F:GAACCAGAACTCCGAGAC	(TGC)5	56	175–190
	R:ATTATCCGCCAGCATATAGT			
SHCSSR7	F:ATGTAGCATCATCAGGAAGA	(TTG)4	56	129–168
	R:ACCCACCAAGTGTTTGAC			
SHCSSR8	F:ATGGTGAATCAGTTTGGTAC	(GT)11 (GA)8	56	129–378
	R:ATGGCGTCTATATTGGAAGT			
SHCSSR10	F:CGACATAAACCCACACATAT	(TC)11	56	120–200
	R:TCTTCAACTCTGACAGTGAT			
SHCSSR11	F:GAAGATGACGACGGAACA	(TCA)4	56	110–216
	R:GGCTACCAATCCTCCATT			
SHCSSR12	F:AAACTGGCTCCCCCGTCT	(AAC)6 (AAG)5	56	100–130
	R:CGGAACCCAAGCATTCCAAAG			
SHCSSR16	F:ACCTCTCCCACAAAATGCACA	(CA)7 (TC)9	56	140–255
	R:ATCGGAGAGCTTGATCAGAAGG			

### Morphological data analysis

We employed SPSS 26 for conducting one-way analysis of variance (ANOVA) to preliminarily assess phenotypic traits across various regions. Subsequently, the phenotypic clustering of *G*. *littoralis subsp*. *littoralis* was examined utilizing the "pheatmap" package in R software [33]. Additionally, we explored the relationship between the major cultivation areas of *G*. *littoralis subsp*. *littoralis* by employing morphological data and utilizing the "pairs panels" function provided by the "psych" R package [34]. Mixed linear models were constructed, and genetic power was calculated using the R package "lme4" [35].

### Genetic diversity and population structure analysis

Various genetic diversity parameters, including the number of alleles (Na), heterozygosity (Ho), Nei’s genetic distance (GD), analysis of molecular variance (AMOVA), and principal coordinate analysis (PCoA), were calculated using GenAlEx 6.5 software [36]. Nei’s genetic diversity index (h), Shannon information index (I), population differentiation index (Fst), gene flow (Nm) were calculated using popgene32 software [37]. Polymorphic information content (PIC) was also determined using PowerMarker (v.3.25) [38]. For constructing phylogenetic trees, the UPGMA (Unweighted Pair Group Method with Arithmetic Mean) and Neighbor-Joining clustering analysis methods were employed based on Nei’s genetic distance of populations using MEGA X software [39]. Additionally, we assessed the correlation between genetic distance and geographic distance using the Mantel test with GenAlEx 6.5 [36]. For Bayesian clustering analysis, STRUCTURE 2.3.4 software was utilized on the 16 populations [40, 41]. We set K values ranging from 2 to 16, with 10 repetitions for each K value, and the Markov chain Monte Carlo (MCMC) was set to 200,000. Structure output files were uploaded to Structure Harvester online to determine the optimal K value for the 16 populations [42]. Cluster repetition sampling analysis was performed using CLUMMP [43], followed by generation of STRUCTURE genetic structure plots using distract1.1 [44].

## Results and analysis

### The correlation and heritability analysis of morphological traits in *G*. *littoralis subsp*. *littoralis*

**Fig 2:**
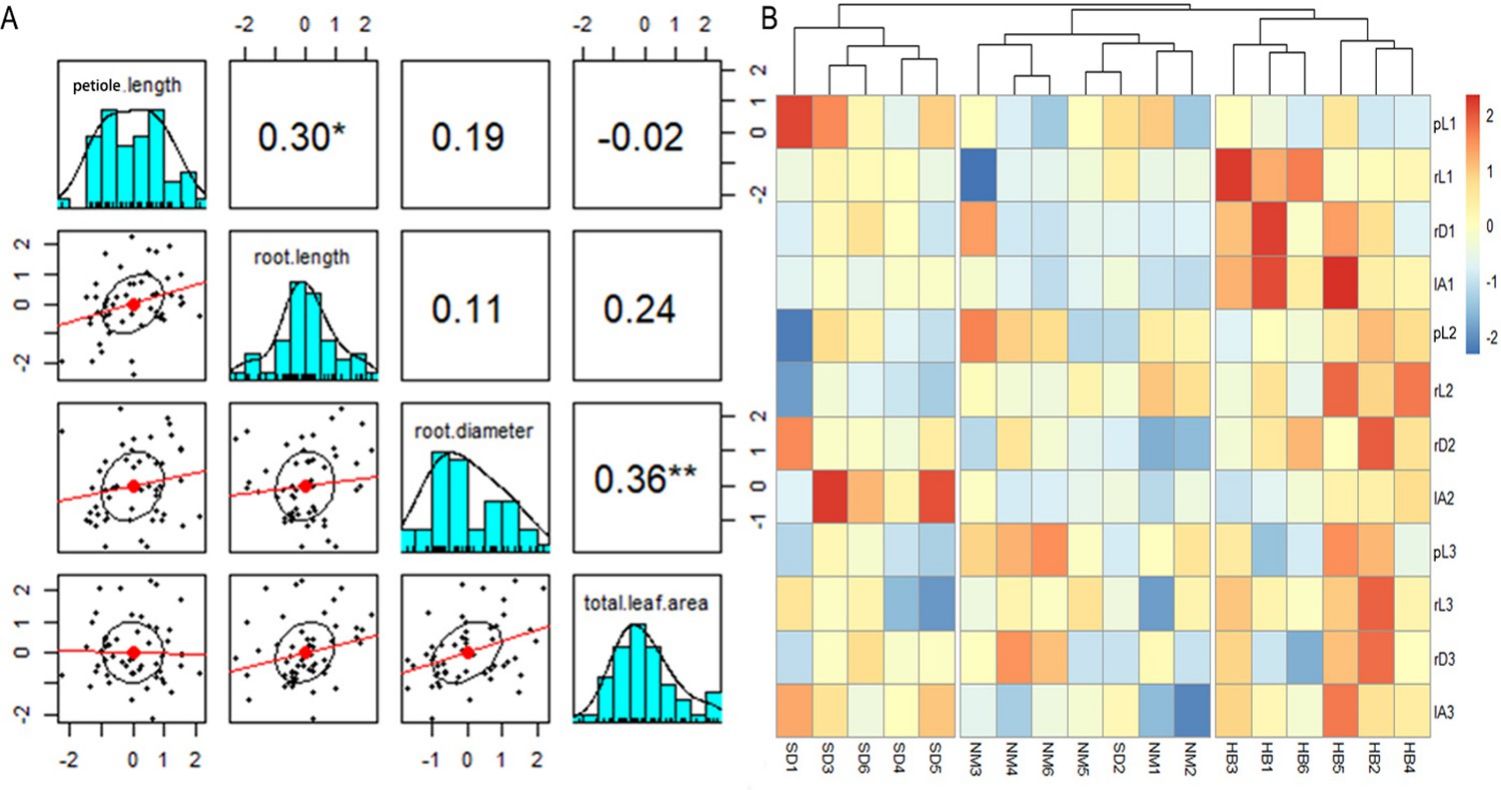
Morphological data analysis. (A) Pearson’s correlation coefficients among morphological data of *Glehnia littoralis subsp*. *littoralis*; (B) Clustering heatmap based on morphological data of *Glehnia littoralis subsp*. *littoralis*. (A) The Pearson coefficients are shown in the upper right corner, with the * sign representing significance and ** representing highly significant, the Pearson visualization in the lower left corner, and the statistical histogram in the middle; (B) pL, rL, rD, lA, represent petiole length, root length, root diameter and leaf area, respectively, numbers 1, 2, and 3 represent the seedling stage, the vigorous growth stage, and the harvest stage.

Morphological trait data, including petiole length, root length, root diameter, and total leaf area of cultivated populations in *G*. *littoralis subsp*. *littoralis* at various growth stages, were measured across three main production areas: Shandong, Hebei, and Inner Mongolia (S1 Table). One-way analysis of variance results revealed significant differences in petiole length at the seedling and harvesting periods in different regions, root length at the seedling and vigorous growth periods in different regions, root diameter during the vigorous growth period in different regions, and total leaf area across regions during all three periods (S2 Table). To assess the genetic influence on the morphological traits of *G*. *littoralis subsp*. *littoralis*, the heritability of each trait across the three periods was calculated. The heritability of different phenotypes ranged from 0.000 to 0.699 (Table 3), with leaf area and root length showing higher heritability during the seedling periods, root length exhibiting relatively higher heritability during the vigorous growth periods, while petiole length and root length displaying relatively higher heritability during the harvesting periods, suggesting that these phenotypes may be more strongly influenced by genetics. Furthermore, Pearson correlation coefficients were computed between different morphological data (Fig 2A). The results demonstrated a significant positive correlation between petiole length and root length of *G*. *littoralis subsp*. *littoralis*, as well as a highly significant positive correlation between root diameter and total leaf area (Fig 2A). Based on the morphological data of 54 samples, cluster analysis was conducted for *G*. *littoralis subsp*. *littoralis* from different regions. The heatmaps (Fig 2B) indicate that samples from the same region mostly cluster together, effectively distinguishing populations distributed in Hebei, Shandong, and Inner Mongolia.

**Table 3:** Heritability for each morphological data of *G*. *littoralis subsp*. *littoralis*.

Traits	Growth stage	Phenotypic variance	Env-Phe inter variance	Random variance	Heritability
Petiole length	The seedling stage	0.000	0.122	0.387	0.036
Root length	The seedling stage	0.152	0.063	0.363	0.683
Root diameter	The seedling stage	0.000	0.000	0.001	0.001
Leaf area	The seedling stage	8.392	5.656	7.086	0.699
Petiole length	The vigorous growth stage	0.003	1.828	13.719	0.000
Root length	The vigorous growth stage	2.569	9.694	14.619	0.284
Root diameter	The vigorous growth stage	0.004	0.006	0.017	0.017
Leaf area	The vigorous growth stage	2.386	158.021	545.732	0.017
Petiole length	The havesting stage	1.884	1.453	6.386	0.567
Root length	The havesting stage	0.916	1.293	6.478	0.402
Root diameter	The havesting stage	0.000	0.008	0.047	0.005
Leaf area	The havesting stage	5.894	130.869	182.228	0.064

### Transcriptome sequencing, assembly, and SSR marker mining

Transcriptome sequencing was performed using the Illumina HiSeq platform (Annoroad, Beijing, China). A total of 86,152 non-redundant overlapping clusters with an N50 length of 1,174 were obtained. Among the 86,152 identified sequences, a total of 13,979 SSR markers were discovered (Table 4). Dinucleotide repeats were found to be the most common with the largest number and proportion (11,544, 82.58%), followed by trinucleotide repeats (2,110, 15.09%), tetranucleotide repeats (207, 1.48%), pentanucleotide repeats (67, 0.48%), and hexanucleotide repeats (52, 0.37%) (Table 4). The number of repeat motifs in SSRs ranged from 5 to 45. The most abundant repeat motif was dinucleotide motifs with five repeats (5,855), followed by dinucleotide motifs with six repeats (2,154), dinucleotide motifs with seven repeats (1,182), and trinucleotide motifs with five repeats (1,069). Furthermore, the frequency distribution of major dinucleotide and trinucleotide motifs was evaluated. The GA/CT motif with five repeats (3,195, 21.13%) was the most common repeat motif in dinucleotide SSRs, while the AAG/CTT motif with five repeats (1,039, 1.58%) was the most common repeat motif in trinucleotide SSRs.

**Table 4:** Summary of SSRs identified in *Glehnia littoralis subsp*. *littoralis*.

Items	Number
Total number of sequences examined	86152
Total size of examined sequences (bp)	73079520
Total number of identified SSRs	13979
Number of SSR containing sequences	10216
Number of sequences containing more than 1 SSR	2556
Number of SSRs present in compound formation	2709
Dinucleotide	11544
Trinucleotide	2110
Tetranucleotide	207
Pentanucleotide	67
Hexanucleotide	52

### Polymorphism analysis of SSR markers

To evaluate the genetic diversity of *G*. *littoralis subsp*. *littoralis*, 16 primer pairs were randomly selected and designed. Each primer successfully generated bands, but six primers (37.5%) showed no polymorphism, while the remaining 10 primers (62.5%) produced polymorphic bands. Finally, amplification was performed using all samples from the 16 *G*. *littoralis subsp*. *littoralis* populations with the 10 primer pairs that generated polymorphic bands. All primers demonstrated a significant level of polymorphism, with the number of alleles (Na) ranging from 2 to 10. When employing 10 pairs of primers to analyze 231 genomic DNAs, a total of 48 bands were identified, out of which 45 were found to be polymorphic, constituting 93.75% of the total bands. On average, each primer amplified 4.8 loci, with 4.5 of them being polymorphic. The calculated parameters including Na, I, Ho, He, and PIC are detailed in Table 5. Notably, PIC is a critical indicator for assessing the efficacy of SSR markers. In this investigation, PIC values ranged from 0.105 (SSR12) to 0.782 (SSR16), with a mean value of 0.419. Among these, three SSR loci (30%) exhibited high information content (PIC > 0.5), five SSR loci (50%) demonstrated moderate information content (0.5 > PIC > 0.25), and only two SSR loci (20%) had low information content (PIC < 0.25) [45]. This suggests that the designed polymorphic SSR markers are suitable for analyzing the genetic diversity of *G*. *littoralis subsp*. *littoralis*.

**Table 5:** Polymorphic information of 10 SSRs in 16 population of *Glehnia littoralis subsp*. ***littoralis*.** Na, Number of alleles; I, Shannon’s Information index; Ho, Observed heterozygosity; He, Expected heterozygosity; PIC, Polymorphism Information Content.

Locus	Na	I	Ho	He	PIC
SHCSSR1	5	0.795	0.263	0.417	0.402
SHCSSR2	4	0.837	0.451	0.451	0.435
SHCSSR3	4	0.944	0.691	0.565	0.529
SHCSSR5	2	0.672	0.839	0.480	0.369
SHCSSR7	3	0.391	0.242	0.222	0.199
SHCSSR8	6	0.592	0.359	0.322	0.309
SHCSSR10	8	1.509	0.459	0.708	0.752
SHCSSR11	2	0.567	0.542	0.382	0.308
SHCSSR12	4	0.191	0.103	0.094	0.105
SHCSSR16	10	1.612	0.813	0.758	0.782
Summary	4.8	0.811	0.476	0.440	0.419

### Genetic diversity and genetic differentiation of *G*. *littoralis subsp*. *littoralis* populations

The genetic diversity of the *G*. *littoralis subsp*. *littoralis* populations is at a relatively high level (h = 0.439, I = 0.811) (Table 6). All 16 populations had relatively high genetic diversity. Overall, the population displaying the highest genetic diversity is the ZS wild population in Zhoushan, Zhejiang (h = 0.531, I = 0.937), while the population with the lowest genetic diversity is the cultivated population C in Shenze, Hebei (h = 0.398, I = 0.752) (Table 6). Among the wild populations, the YT and RZ populations in Shandong exhibit lower genetic diversity, while the CH, WF, ZH, and XC populations in Liaoning demonstrate moderate diversity. The cultivated populations in Shandong exhibit the highest genetic diversity (h = 0.441, I = 0.876), whereas the genetic diversity of cultivated populations in Inner Mongolia (h = 0.410, I = 0.741) is relatively lower. Comparatively, wild populations generally exhibit higher genetic diversity levels than cultivated ones (Cultivated populations: h = 0.441, I = 0.877; Wild populations: h = 0.491, I = 0.930). Overall, the genetic diversity of the *G*. *littoralis subsp*. *littoralis* populations is at a relatively high level (h = 0.439, I = 0.811).

**Table 6:** Population genetic diversity statistics and genetic differentiation. I, Shannon’s Information index; Ho, Observed heterozygosity; *h*, Nei’s (1973) gene diversity.

Populations	I	Ho	h
Cultivated populations	0.877	0.454	0.441
HB cultivated population	0.873	0.464	0.440
A	0.834	0.477	0.489
B	0.873	0.533	0.459
C	0.752	0.421	0.398
D	0.773	0.444	0.419
E	0.795	0.477	0.424
F	0.876	0.441	0.442
SD cultivated population	0.876	0.430	0.441
G	0.819	0.456	0.432
H	0.844	0.405	0.432
NM cultivated population	0.741	0.433	0.410
I	0.741	0.433	0.410
Wild populations	0.930	0.508	0.491
YT	0.759	0.470	0.429
RZ	0.737	0.456	0.428
CH	0.830	0.470	0.439
WF	0.772	0.527	0.439
ZH	0.808	0.560	0.459
XC	0.827	0.471	0.465
ZS	0.937	0.580	0.531
Total	0.811	0.476	0.439

Note: HB represents the population of Hebei Province, SD represents the population of Shandong Province, NM represents the population of Inner Mongolia.

To assess within- and among-population genetic differentiation, an analysis of molecular variance (AMOVA) was conducted. The 16 *G*. *littoralis subsp*. *littoralis* populations were categorized into cultivated, wild, and total populations based on their sources. Key indicators including genetic variation index (Fst), gene flow (Nm), and percentage of variation were calculated (Table 7). The results reveal that genetic differentiation between cultivated populations (Fst = 0.030) is lower than that between wild populations (Fst = 0.072). Moreover, gene flow between cultivated populations (Nm = 7.973) surpasses that between wild populations (Nm = 3.234). Within cultivated populations, the variation rate between populations is 0.67%, while the variation rate within populations is 99.32%. Conversely, within wild populations, the variation rate between populations is 3.37%, and the variation rate within populations is 96.62%. Considering all populations, the variation rate between populations of *G*. *littoralis subsp*. *littoralis* is 2.28%, while the variation rate within populations is 97.71% (Fst = 0.061, Nm = 3.880). These results indicate that the genetic variation of wild populations and cultivated populations of *G*. *littoralis subsp*. *littoralis* mainly comes from within populations rather than between populations.

**Table 7:** Analysis of molecular variance (AMOVA) of *Glehnia littoralis subsp*. *littoralis* populations. Fst, genetic differentiation index; Nm, gene flow.

Sources of Variation	Degrees of Freedom	Sum of Squares	Mean Square	Estimated Variance	Percentage of variation	Fst	Nm
**Cultivated populations**						0.030	7.973
Among populations	8	21.505	2.688	0.016	0.67%		
Within Individuals	319	701.553	2.268	2.268	99.32%		
Total	327	723.058	-	2.284	100%		
**Wild populations**						0.072	3.234
Among populations	6	23.671	3.945	0.089	3.37%		
Within Individuals	127	305.15	2.537	2.537	96.62%		
Total	133	328.821	-	2.626	100%		
**All populations**						0.061	3.880
Among populations	15	55.997	3.733	0.055	2.28%		
Within Individuals	446	1006.702	2.346	2.346	97.71%		
Total	461	1062.699	-	2.401	100%		

**Fig 3:**
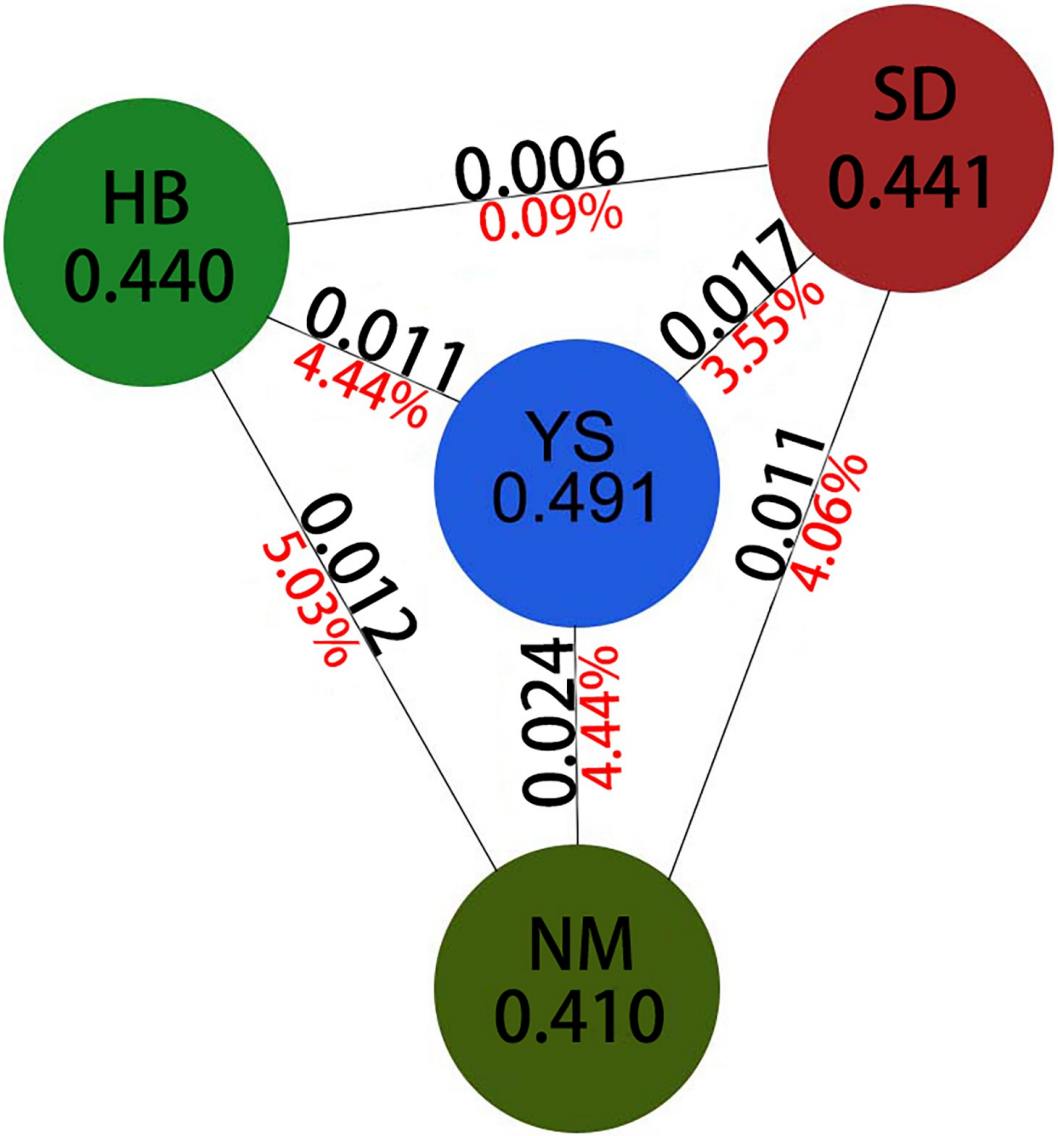
Analysis of genetic diversity and genetic differentiation indices between cultivated and wild populations of *Glehnia littoralis subsp*. *littoralis*. Values in circles are Nei’s genetic diversity. Black numbers (above) are Fst between two connected populations, and red numbers (below) are percent differences between two connected populations by the AMOVA analysis. HB represents the population of Hebei Province, YS represents the wild population, SD represents the population of Shandong Province, NM represents the population of Inner Mongolia.

To gain a deeper understanding of the genetic differentiation relationship between cultivated and wild populations of *G*. *littoralis subsp*. *littoralis* in different cultivation regions, the 16 *G*. *littoralis subsp*. *littoralis* populations underwent further categorization into Shandong cultivated populations, Hebei cultivated populations, Inner Mongolia cultivated populations, and wild populations. The Nei’s genetic diversity (h) values for the Shandong cultivated population, Hebei cultivated population, and Inner Mongolia cultivated population were determined to be 0.441, 0.440, and 0.410, respectively (Table 6). Notably, the Shandong cultivated population exhibited relatively higher genetic diversity compared to the other cultivated populations, although no distinct advantages were observed. The genetic differentiation coefficient (Fst) provided insightful patterns regarding the genetic relationships among different populations of *G*. *littoralis subsp*. *littoralis*. In the comparison between Hebei and Inner Mongolia populations, the Fst value of 0.012 indicated relatively high genetic differences, accompanied by a variation (Va) of 5.03% (Fig 3). Similarly, the Fst value of 0.011 between the Shandong and Inner Mongolia populations suggested a pronounced genetic divergence, with a variation of 4.06% (Fig 3). In contrast, the Fst value of 0.006 between the Shandong and Hebei populations indicated a relatively similar genetic composition, with only 0.09% variation (Fig 3). Considering the context of cultivated and wild populations, the Fst value of 0.024 between the wild and Inner Mongolia cultivated populations implied relatively high genetic differences, with a variation of 4.44% (Fig 3). The Fst value of 0.017 between the Shandong cultivated and wild populations revealed mild genetic distinctions, accompanied by a variation of 3.55% (Fig 3). Additionally, the Fst value of 0.011 between the Hebei cultivated and wild populations indicated slight genetic differentiation, with a variation of 4.44% (Fig 3). Overall, all these findings revealed relatively low levels of genetic differentiation across the studied populations.

Among these populations, those cultivated in Hebei and Inner Mongolia exhibit the highest degree of genetic differentiation, whereas the populations in Hebei and Shandong display the lowest level of genetic variance. Analysis of the genetic differentiation between the wild population and the populations in the three cultivation regions reveals that the cultivated population in Inner Mongolia shows the greatest genetic differentiation from the wild population, followed by Shandong, and Hebei exhibiting the least differentiation. These findings illuminate the genetic distinctions and diversity dynamics within and between these populations. Overall, both the wild and cultivated populations of *G*. *littoralis subsp*. *littoralis* demonstrate a high level of genetic diversity. The cultivated population in Inner Mongolia exhibits the greatest differentiation from both the cultivated populations in Shandong and Hebei, as well as from the wild populations. Nonetheless, the differentiation among all cultivated populations remains relatively low, with population variation primarily originating from within each population.

### Genetic structure analysis of *G*. *littoralis subsp*. *littoralis* populations

**Fig 4:**
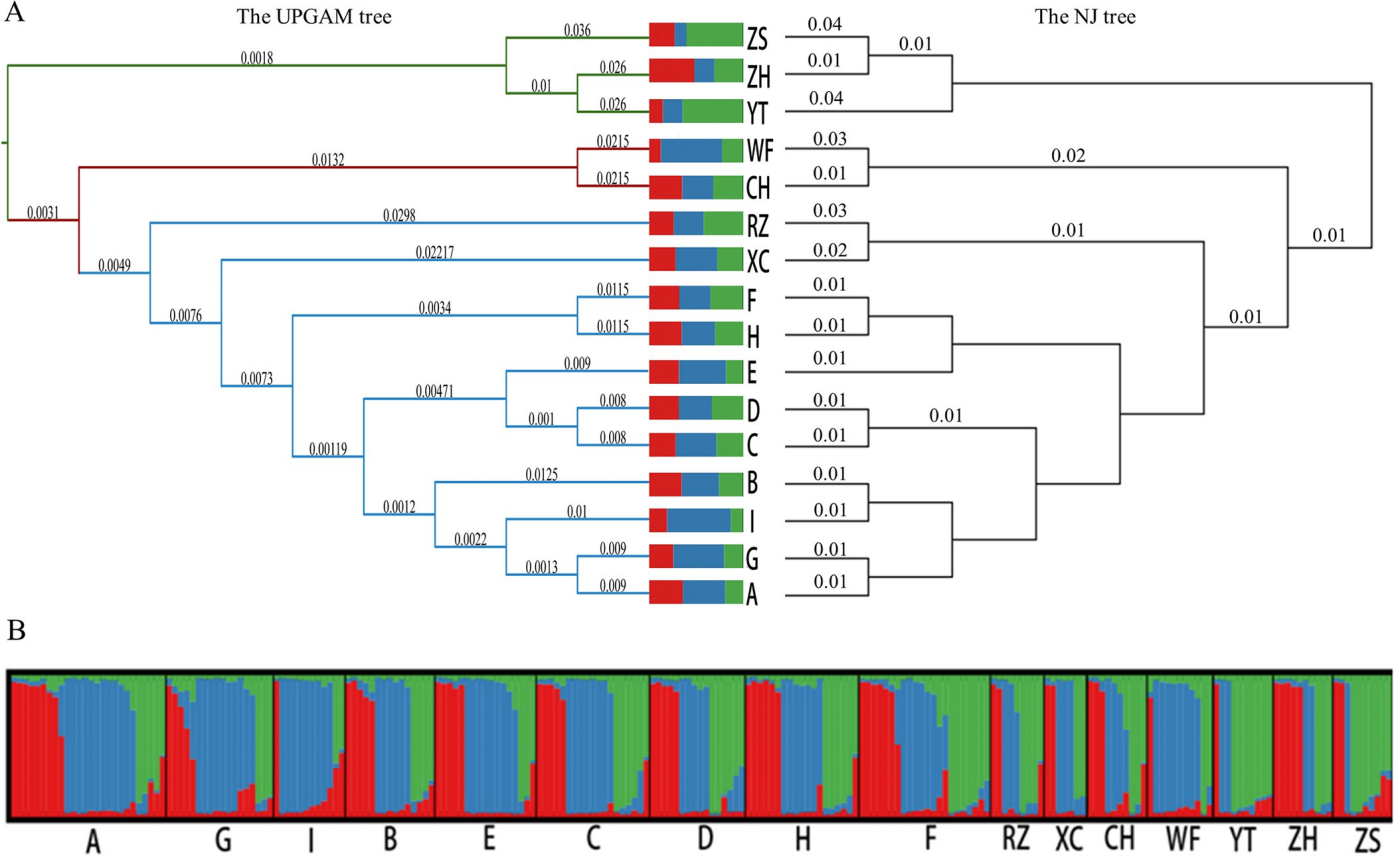
Genetic structure analysis of 16 populations. (A) The phylogenetic tree of the *Glehnia littoralis subsp*. *littoralis* population based on the UPGMA and NJ methods. (B) The genetic structure of populations in three colors assigned by STRUCTUR software.

**Fig 5:**
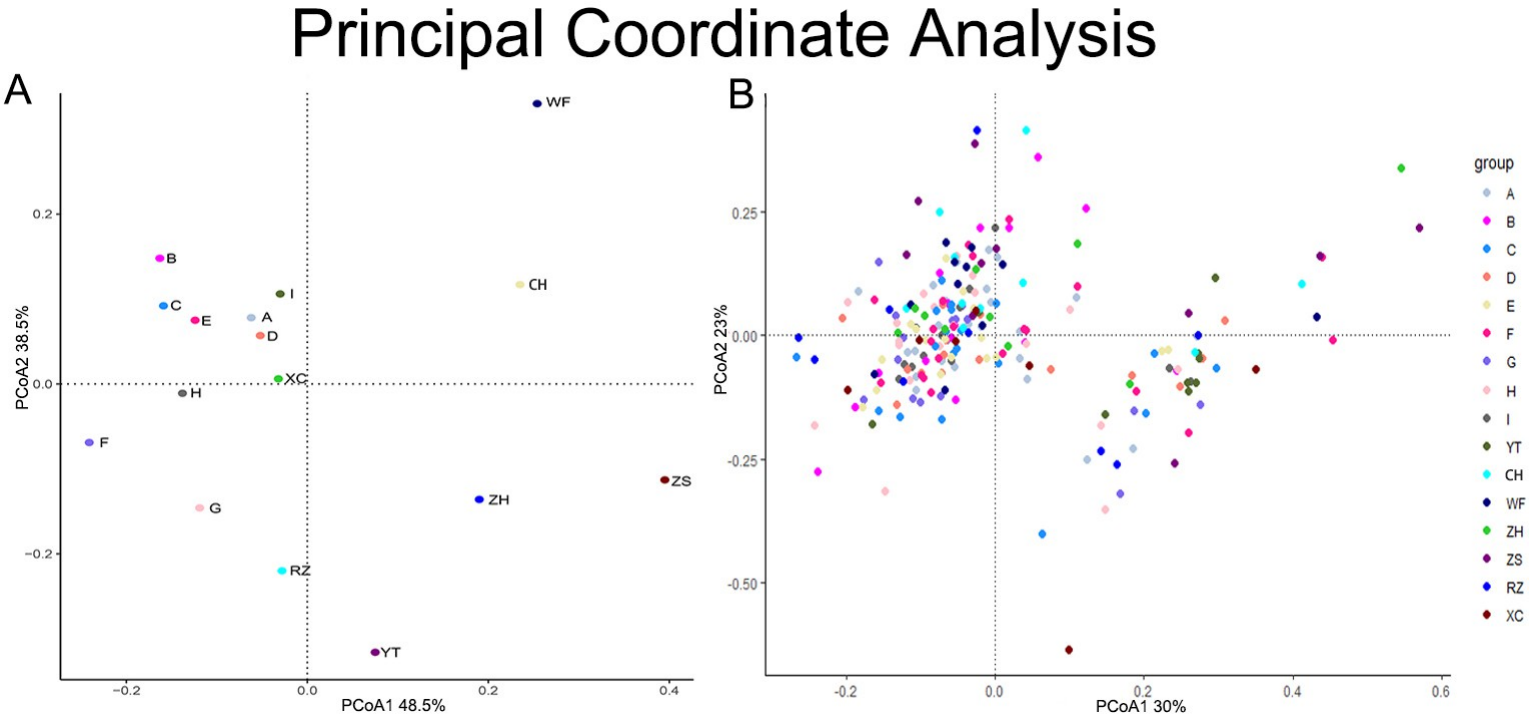
(A) Principal Coordinate Analysis (PcoA) for 16 populations of *Glehnia littoralis subsp*. *littoralis* and (B) PcoA for all individuals of 16 populations.

Utilizing the genetic distance matrix of the 16 populations, the UPGMA and NJ methods were employed to construct phylogenetic trees for *G*. *littoralis subsp*. *littoralis* (Fig 4A). The 16 populations were predominantly categorized into three branches. Branch 1 encompasses all nine cultivated populations A-I from the cultivation regions of Hebei, Shandong, and Inner Mongolia, as well as two wild populations RZ and XC from Shandong and Liaoning. Within this branch, the populations A, B, and G in Hebei, along with the I populations in Inner Mongolia, form a distinct subgroup; the C, D, and E populations in Hebei cluster into a separate subgroup; while the H and F populations in Shandong and Hebei converge into another subgroup. Branch 2 encompasses two wild populations, WF and CH, originating from Dalian and Wafangdian in Liaoning, respectively. Branch 3 comprises the remaining three wild populations, YT, ZS, and ZH, sourced from Shandong, Zhejiang, and Liaoning, respectively. To delve deeper into the genetic structure of each population, we conducted a structural analysis using the model-based Bayesian clustering software STRUCTURE (Fig 4B). Analysis of K values (number of clusters) ranging from 1 to 16 revealed that ΔK peaked at K = 3, indicating no tendency to further subdivide into additional subgroups, suggesting the presence of three clusters among these 16 populations. A Principal Coordinate Analysis (PCoA) was performed to further elucidate the genetic structure of *G*. *littoralis subsp*. *littoralis* (Fig 5A). The two axes accounted for 48.5% and 38.5% of the genetic variation, respectively, totalling 87%. The PCoA manifested a pronounced topological distribution, with the nine cultivar populations (A-I) forming a cohesive cluster and demonstrating closer genetic distances to the wild populations XC and RZ. This pattern underscores a profound genetic affinity between these groups. The remaining five wild populations, WF, RZ, ZH, ZS, and YT, exhibited a stronger genetic relationship, with WF and CH showing closer genetic distances, while YT, ZH, and ZS showed closer genetic distances. These observations align with the outcomes of the clustering analysis. To dissect the individual contributions to the genetic architecture, a PCoA was also executed on a per-individual basis. Here, the genetic variations explained by the two axes were 30% and 23%, aggregating to 53% (Fig 5B). Interestingly, the individuals of *G*. *littoralis subsp*. *littoralis* did not cluster neatly according to their populations. Instead, they were interspersed in a somewhat chaotic pattern, yet a broad bifurcation into two clusters could be discerned (Fig 5B).

The Mantel test served as a tool to probe the correlation between genetic and geographic distances within *G*. *littoralis subsp*. *littoralis*, offering insights into the spatial genetic dynamics of the species. A statistically significant positive correlation between Nei’s genetic distance and the geographic distance among wild populations (R = 0.1178, *P* = 0.014), while no significant correlation was discerned between Nei’s genetic distance and the geographic distance among cultivated populations (R = 0.01, *P* = 0.44).

## Discussion

Typically, the genetic diversity of crops undergoes alterations due to artificial cultivation and domestication, manifesting as consequences of artificial selection, founder effects, and genetic bottlenecks [46–48]. In the early phases of agricultural practices, a restricted number of wild individuals are chosen for cultivation, resulting in the dissemination of genes from the selected wild specimens into the cultivated offspring [49, 50]. Throughout cultivation, genetic diversity undergoes further scrutiny, favoring individuals with desirable growth traits and robust roots. Consequently, this process contributes to a subsequent reduction in genetic diversity. Numerous studies have illustrated that the genetic diversity of cultivated varieties is significantly diminished compared to their wild counterparts, the phenomenon observed in various crops such as corn, rice, soybeans, and medicinal plants [51–53]. Our investigation aligns with these findings, demonstrating a lower genetic diversity in the cultivated population of *G*. *littoralis subsp*. *littoralis* compared to its wild counterpart (h = 0.441 in cultivated population, h = 0.491 in wild population). Notably, the decline in genetic diversity within the cultivated population of *G*. *littoralis subsp*. *littoralis* is limited, with relatively high genetic diversity preserved at the DNA level. In some medicinal plants that have been cultivated for a relatively short period of time, cultivated populations have also been found to pass through a slight genetic bottleneck, such as *Scutellaria baicalensis* Georgi [54], *Gastrodia elata* Blume [55] and Huang-lian [56]. This preservation of higher genetic diversity in cultivated population of *G*. *littoralis subsp*. *littoralis* may be attributed to the relatively short domestication period of medicinal plants. Moreover, during the widespread cultivation of medicinal plants, there is also gene exchange with wild populations, mitigating the rate of decline in genetic diversity in comparison to crops [50, 57, 58]. Gene flow, being a determinant of genetic structure, plays a crucial role in shaping population dynamics. In small populations, genetic drift tends to fix specific genotypes, thereby diminishing overall genetic diversity. However, in situations where gene flow between populations is substantial, the impact of genetic drift is mitigated [58]. In addition, we hypothesize that the continued occurrence of outcrossing sexual reproduction between cultivation populations and wild populations of *G*. *littoralis subsp*. *littoralis* along coastal regions where human activity, illegal transplanting and seed collection of wild germplasm resources increase the gene exchange between the wild and cultivated populations and enrich the gene pool, which resisted the sharp decline of genetic diversity in the cultivated population of *G*. *littoralis subsp*. *littoralis*. The observed higher gene flow between cultivated and wild populations (Nm = 3.880) lends further support to this hypothesis.

Despite the geographical distance separating the three cultivation regions (Hebei, Shandong, and Inner Mongolia), analysis reveals a noteworthy level of genetic diversity within the cultivated population, with minimal genetic differentiation observed among these regions (Fst = 0.030). This is much lower than the genetic differentiation coefficient among some other cultivated populations of medicinal materials. For example, the average genetic differentiation coefficient among populations of non-crop cultivars is 0.212 [59], while the genetic differentiation coefficient among cultivated populations of *S*. *baicalensis* is 0.220 [54]. This phenomenon can be attributed to the considerable gene flow detected between *G*. *littoralis subsp*. *littoralis* populations (Nm = 7.973), facilitated by seed exchange within the market, which fosters genetic homogenization within the cultivated population. For medicinal plants cultivated in China, mixed cultivation of seeds from different geographical locations is a common practice. In addition, long-term artificial selection and intra-varietal inbreeding imposed by plant breeding may lead to widespread genetic variation among cultivated cultivars [46]. However, the farmers who planted Radix Glehniae did not distinguish the cultivated species in the process of planting, so it is unlikely to promote genetic differentiation among cultivated populations. In recent years, the cultivation in Shandong has decreased, raising concerns about its genetic status. However, it’s noteworthy that the genetic diversity assessments from three cultivation areas indicate no significant differences among them (Table 6). Despite this, Shandong’s cultivated population exhibits relatively high genetic diversity, underscoring its continued genetic importance.

There exist two primary hypotheses concerning the origin of crop cultivation: single origin and multiple origins [60–62]. The single origin hypothesis suggests a domestication process wherein the cultivated population is continuously transplanted from one limited area to other regions. Conversely, multiple origins entail the cultivation of a species through multiple introductions from its wild ancestors across its entire distribution range. Phylogenetic tree analysis using SSR markers revealed that all nine cultivated populations of *G*. *littoralis subsp*. *littoralis* clustered together, suggesting a potential single origin of domestication across primary planting areas. Wild populations RZ and XC from Shandong and Liaoning showed a close relationship, consistent with previous molecular findings [20, 63]. These wild populations grouped with cultivated ones on the phylogenetic tree, indicating a single domestication origin, likely from Rizhao in Shandong and Xingcheng in Liaoning, or a common, yet unsampled ancestor. The results of PCoA and structural analysis revealing the genetic composition also support this assumption (Figs 4 and 5). However, due to limited sampling, the exact domestication origin remains uncertain. Based on the analysis of genetic differentiation among cultivated populations, it is evident that populations in Inner Mongolia exhibit the highest degree of differentiation compared to cultivated populations in Hebei and Shandong regions and wild populations (Fig 3). Conversely, the genetic differentiation between populations in Hebei and Shandong is relatively minimal. Considering Shandong as the earliest recorded area for cultivation, it is hypothesized that the cultivated population in Shandong originated earliest and subsequently spread to Hebei and Inner Mongolia. Proximity between Shandong and Hebei geographically can contribute to slight genetic variations between them. Mantel testing results reinforce this observation: genetic distance (Nei’s) in wild populations positively correlates significantly with geographic distance (R = 0.1178, *P* = 0.014), while in cultivated populations, the correlation is positive but not statistically significant (R = 0.01, *P* = 0.44).

Traditional agriculture is considered to be an important reservoir of genetic variation [64, 65]. The introduction of wild populations in different locations can broaden the genetic background of cultivated populations and is an effective way to maintain and protect the gene pool of species. Although our results show that the cultivated species have high genetic diversity, traditional cultivation can be used as an effective strategy to protect its genetic resources. However, the cultivation process may be accompanied by the loss of some rare alleles, resulting in the loss of important haplotypes of the species, which has been observed in the cultivation process of *S*. *baicalensis* [54, 66]. In genetic conservation programs, rare alleles are considered as minor factors, but they are important for long-term evolution or for achieving new breeding goals, such as resistance to introduced insects or diseases [66]. Therefore, in the absence of sufficient genetic information to prove that important alleles in cultivated populations are effectively preserved, wild resources are still irreplaceable.

Traditional Chinese medicine industry in China is growing rapidly, and the demand for traditional Chinese medicine is on the rise. Due to the gradual depletion of wild *G*. *littoralis subsp*. *littoralis* resources, it is facing the threat of extinction [15] and can no longer be used. Therefore, the cultivation yield and quality of *G*. *littoralis subsp*. *littoralis* urgently need to be improved. Achieving high yield and quality mainly involves two key factors: the selection of good cultivars and the implementation of effective field management measures. At present, the seeds used for planting cultivated *G*. *littoralis subsp*. *littoralis* are mainly from cultivated populations. The availability of mixed seed sources on the market is considered to be an important factor leading to the unstable quality of medicinal materials. In order to ensure the production of high-quality cultivars, it is very necessary to select the seed source of excellent varieties manually. Preventing seed mixing, establishing artificial seed production base and reducing the use of inferior seeds are the key factors affecting yield. In addition, field management is also an important step to ensure high yield. The phenotypic data analysis collected in this study (Fig 2) indicates a significant correlation between root traits and leaf area as well as petiole length. This correlation underscores the importance of monitoring petiole length and leaf area during cultivation processes. Such observations aid in assessing the developmental status of plant roots and can facilitate production management decisions aimed at enhancing yield. Hence, they serve as intuitive indicators providing information for on-site management practices. Genetic heritability analysis of the phenotypic data collected in this study (Table 3) reveals that root length, leaf area, and petiole length maintain relatively high heritability across three growth stages. This observation suggests that these phenotypes are primarily influenced by genetic factors. Integrating these findings with genetic analysis of the target species further provides a foundational theoretical basis for the selection, breeding, and propagation of its superior subspecies. Additionally, the heritability analysis (Table 3) also reveals that morphological traits are not solely determined by genetic factors. Specifically, during growth stages, only root length shows moderate correlation with heritability, indicating its influence not only by genetic factors but also by other elements such as microbial interactions and soil properties [67]. Therefore, these influencing factors must be considered throughout the entire cultivation process to optimize management outcomes.

## Conclusion

This study employed 10 SSR markers to genotype 164 cultivated and 67 wild samples of *G*. *littoralis subsp*. *littoralis*, conducting phenotypic analysis on 54 cultivated specimens. SSR markers indicated high overall genetic diversity, with cultivated populations showing slightly lower diversity compared to their wild counterparts. Despite this, there was minimal genetic differentiation between cultivated and wild populations, supported by frequent gene flow. Phylogenetic analysis pointed to a probable single origin of domestication, likely in Rizhao, Xingcheng, or their common ancestor region. Heritability analysis underscored genetics as the primary influence on root length, possibly modulated by environmental factors. Pearson correlation coefficients highlighted significant positive relationships between petiole length and root length, as well as between root diameter and total leaf area. This study provides foundational insights for genetic enhancement, molecular marker-assisted breeding, and field management strategies for *G*. *littoralis subsp*. *littoralis*. A limitation is our focus on a limited number of phenotypic traits and a small sample size, restricting a comprehensive analysis of factors influencing the yield of medicinal materials. Moreover, due to the small sample size, we were unable to pinpoint the exact single ancestral source of cultivated populations. Future research should expand sample sizes to address the shortcomings of this study.

## Supporting information

S1 Table The phenotypic data of cultivated *G*. *littoralis subsp*. *littoralis*. (XLSX)

S2 Table ANOVA analysis of the phenotype of *G*. *littoralis subsp*. *littoralis* in different regions. (XLSX)
